# Scoring Abdominal Symptoms in People with Cystic Fibrosis

**DOI:** 10.3390/jcm13061650

**Published:** 2024-03-13

**Authors:** Harold Tabori, Anton Barucha, Carlos Zagoya, Franziska Duckstein, Gabor A. Dunay, Pauline Sadrieh, Louise Polte, Jochen G. Mainz

**Affiliations:** 1 Cystic Fibrosis Centre, Brandenburg Medical School (MHB) University, Klinikum Westbrandenburg, 14770 Brandenburg an der Havel, Germany; 2 Alexianer St. Hedwig Kliniken Berlin GmbH Hospital, Gastroenterology, Internal Medicine, 12526 Berlin, Germany; 3 Faculty of Health Sciences, Joint Faculty of the Brandenburg University of Technology Cottbus-Senftenberg, the Brandenburg Medical School Theodor Fontane and the University of Potsdam, 14476 Potsdam, Germany; gabor.dunay@uk-brandenburg.de; 4 Brandenburg Medical School (MHB), University, Gastroenterology, Internal Medicine, 16816 Brandenburg an der Havel, Germany; 5 Brandenburg Medical School (MHB), University, Pediatric Gastroenterology, Klinikum Westbrandenburg, 16816 Brandenburg an der Havel, Germany

**Keywords:** gastrointestinal, patient-reported outcome measure (PROM), CFTR, modulator, PedsQL-GI, PAGI-SYM, PAC-SYM, PAC-QOL, CFAbd-Score

## Abstract

(1) Background: The introduction of highly effective CFTR-modulating therapies (HEMT) has changed the course of the disease for many people with Cystic Fibrosis (pwCF). Attention previously focused on life-threatening conditions of the respiratory system has broadened, bringing the involvement of the digestive system into the clinical and scientific focus. This emphasized the need for sensitive tools to capture and quantify changes in abdominal symptoms (AS), ideally applying patient-reported outcome measures (PROMs). (2) Methods: The present review focuses on studies addressing AS assessment deriving from the multi-organic abdominal involvement in pwCF. Among 5224 publications retrieved until Nov. 2022, 88 were eligible, and 39 were finally included. (3) Results: The review reveals that for a long time, especially before HEMT availability, AS in pwCF were assessed by single questions on abdominal complaints or non-validated questionnaires. PROMs focusing on quality of life (QOL) including a few GI-related questions were applied. Likewise, PROMs developed and partially validated for other non-CF GI pathologies, such as chronic inflammatory bowel diseases, irritable bowel syndrome, gastroesophageal reflux, constipation, or pancreatitis, were implemented. (4) Conclusions: Only lately, CF-specific GI-PROMs have been developed and validated following FDA guidelines, showing high sensitivity to changes and capturing marked and statistically significant reductions in the burden of AS achieved with HEMT implementation.

## 1. Introduction

Cystic fibrosis (CF) is the most common life-limiting hereditary disease in the Caucasian population, caused by dysfunction of the CF Transmembrane Conductance Regulator (CFTR), an anion channel lining the epithelial cells throughout the body. At present, more than 2000 mutations in the CFTR gene have been identified, with about 400 being causative for the disease [1,2]. Dysfunction of the CFTR channels leads to impaired fluid homeostasis, causing a multi-organ disease, most relevant in the respiratory and digestive systems. Whereas the respiratory system has been the primary focus in the past decades, as 90% of people with CF (pwCF) died prematurely from pulmonary destruction, the recent improvement in the course of the disease, including highly effective CFTR-modulating therapies (HEMT) availability, has brought the multi-organ abdominal involvement into clinical and scientific focus [3].

**Figure 1:**
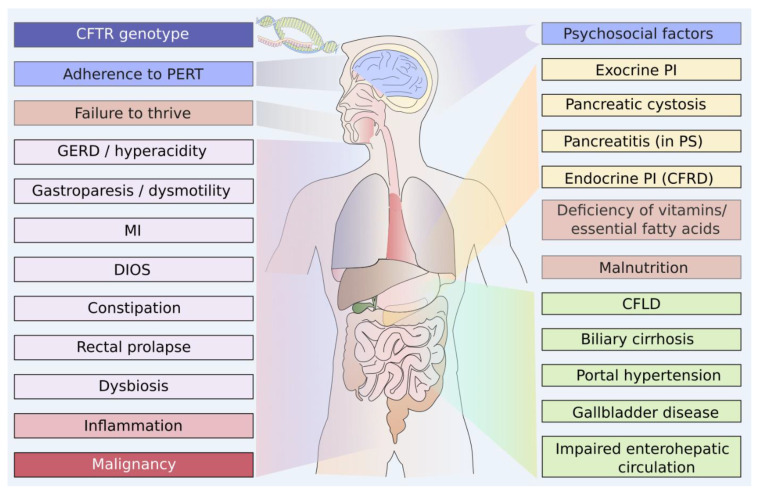
Multifactorial aspects contributing to the burden of gastrointestinal symptoms in pwCF MI = meconium ileus; DIOS: Distal Intestinal Obstruction Syndrome; PERT: Pancreatic Enzyme Replacement Therapy; PI: pancreatic insufficiency, PS: pancreatic sufficiency; GERD: Gastroesophageal Reflux Disease; CF: cystic fibrosis; CFLD: CF-associated Liver Disease, CFRD: CF-Related Diabetes, CFTR: CF Transmembrane Conductance Regulator. Figure adapted from [5].

Multiple aspects, directly or indirectly related to the inherited CFTR disorder in the digestive system, cause the specific pattern of abdominal symptoms (AS) in pwCF, which contributes relevantly to the burden of the disease (Figure 1) [4].

Starting already in utero, pwCF carrying two severe CFTR mutations (class I, II, or III) reveal considerable pancreatic destruction. Viscous pancreatic fluids obstruct small pancreatic ducts and acini causing inflammation, dilatation, fibrosis, fat infiltrations, and ultimately exocrine pancreatic insufficiency (EPI). This condition is prevalent in approximately 85% of pwCF already before the second year of life. In the long run, pancreatic destruction and chronic inflammation may also cause endocrine PI, and CF-related diabetes (CFRD), occurring in about 50% of pwCF above the age of 40 years and affecting relevant proportions of pwCF from the age of 10 years [6]. Untreated EPI results in maldigestion, malabsorption, and failure to thrive, together with deficient absorption of fat-soluble vitamins and other nutrients. Intestinal CFTR deficiency further aggravates enterocyte dysfunction, which breaks the digestive balance leading to stool abnormalities, intestinal obstruction [7], disruption of the intestinal flora [8], and defective bile acid absorption with a specific cholestatic pattern in pwCF [9,10,11,12]. Thereby, CFTR-mRNA expression is highest in the Brunner glands of the proximal duodenum, which produce intestinal mucus and it decreases towards the distal intestine [13,14]. Only minimal amounts of CFTR channels are found in the stomach.

About 10–15% of pwCF experience a meconium ileus (MI) and later in life, distal intestinal obstruction syndrome (DIOS) is a characteristic GI complication of pwCF. However, pwCF suffer more frequently from constipation, which, unlike DIOS, responds well to stool-softening therapies [15]. Furthermore, anal prolapses and intestinal intussusception occur significantly more often in pwCF [7]. The combination of thickened mucus within the gut and altered pH levels due to insufficient liberation of pancreatic bicarbonate contribute to intestinal dysbiosis, small intestinal bacterial overgrowth (SIBO), and intestinal inflammation [16,17]. Frequent antibiotic treatment of pulmonary pathogen colonization and altered intestinal passage times [18] contribute to the CF-specific AS pattern, which is complemented by changes in bile acid profiles, gallstones, and an impaired enterohepatic circuit [19]. The defective CFTR channel in the bile ducts adds to the pathogenesis of CF-related liver disease (CFLD), initially featuring a toxic cholestatic pattern [9] and progressing to focal or multifocal biliary cirrhosis and end-stage liver cirrhosis that contributes to the appearance of other complications such as portal hypertension, ascites, esophageal varices, and in some cases to liver failure [20].

The resulting broad spectrum of AS may include upper and lower gastrointestinal (GI) symptoms, comprising abdominal pain, flatulence, bloating, meteorism, constipation, diarrhea, fatty stools, and other complaints affecting quality of life (QOL) [5]. Gastroesophageal reflux disease (GERD) [21] is commonly characterized by heartburn or a burning sensation behind the sternum, associated with regurgitation of sour chymus and cough [22]. Furthermore, although longer oro-caecal transit times have been observed in pwCF, the concomitantly increased small bowel water content suggests an impaired ileal emptying, rather than gastric emptying [23]. Manifestation of gastroparesis includes symptoms of fullness, bloating, or abdominal pain [21]. Delayed gastrointestinal transit time can also contribute to constipation and DIOS [7].

This concise description of CFTR-related manifestations in different abdominal organs indicates that there is a complex spectrum of related pathologies, which may lead to a specific burden of AS in pwCF. Accordingly, studies investigating abdominal involvement in pwCF are required to better understand the pathogenesis, pattern, and amelioration of AS. Therefore, diagnostic tools to accurately measure the CF-specific burden of AS and the possible effects of therapeutic interventions are essentially required [12,16]. Since the recent broad availability of HEMT, which in vitro allows for up to 50% reconstitution of CFTR function [24], it has become increasingly relevant to accurately capture and quantify the in vivo effects of this highly effective therapy including patient-reported AS.

## 2. Materials and Methods

We conducted a systematic literature search on PubMed up to 20 November 2022. For the sake of generality, we have used the following search criteria:

(cystic fibrosis OR CF) AND (abdom* OR gastrointestinal OR GI) AND

(symptom* OR pain OR PRO* OR measure OR patient related OR patient reported OR score OR burden)

Reviewed articles were selected in two screening steps. First, two authors independently selected articles according to a title screening with the following criteria:

1. Studies should have included pwCF and
2. Studies should have addressed a research question on gastrointestinal/abdominal symptoms.

In the second screening step, abstracts from articles selected during step one were carefully surveyed. At this stage, the aim was to exclude articles according to the following criteria:

1. Articles not addressing GI symptoms
2. Articles consisting of case reports and reviews.

The resulting articles selected for full-text review were classified into two categories: (1) studies conducted previous to the era of CFTR modulator therapy and (2) studies carried out after approval of the first CFTR modulator ivacaftor in January 2012 to 20 November 2022 [25]. The studies in (2) were further grouped into two groups: (a) studies not focused on assessing HEMT therapies and (b) studies focused on assessing HEMT therapies.

## 3. Results

**Figure 2:**
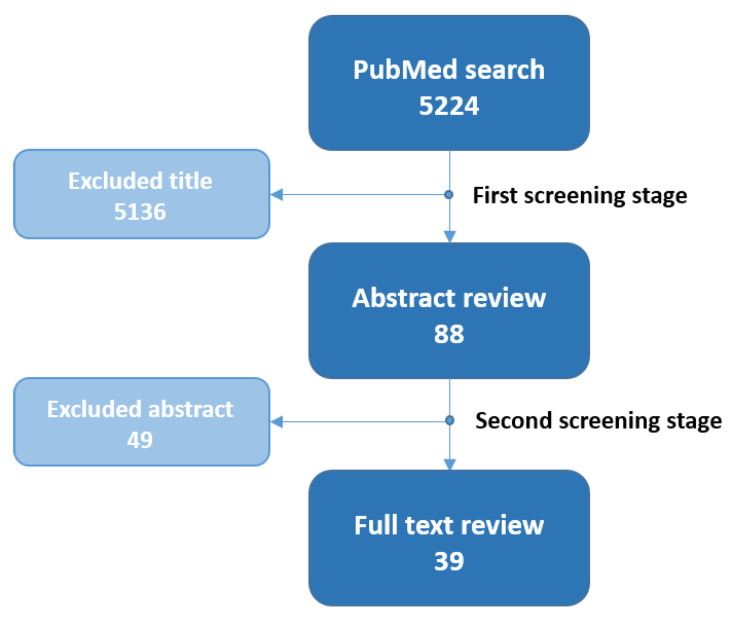
Flow chart regarding literature search and the two-stage review process, whereby a total of 39 suitable articles were selected for full-text review.

The initial search on PubMed yielded a total of 5224 results, of which 88 were selected during the first screening stage described in Section 2. During the second stage, 49 articles were excluded following the criteria stated in Section 2, so that 39 articles were finally included in this review (see Figure 2).

For the sake of clarity, the names of the methods used to capture GI symptoms, e.g., PROMs or non-PROMs (questionnaires or surveys) are highlighted in bold.

### 3.1. Studies Previous to the Era of CFTR-Modulating Therapies

**Figure 3:**
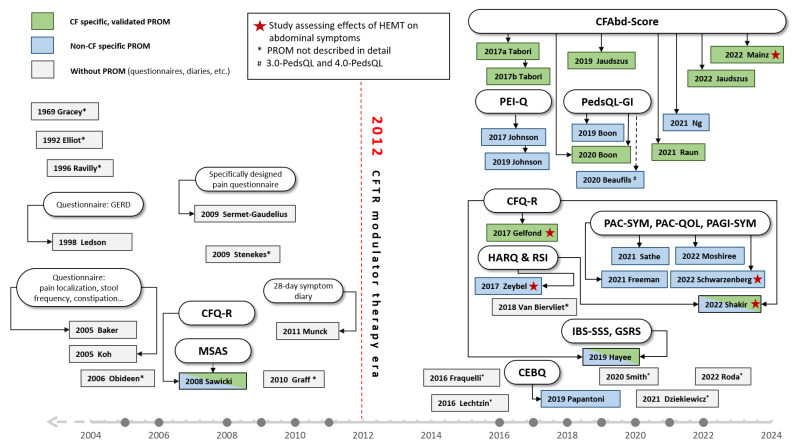
Reviewed studies in the course of the respective year of publication. Red dotted line indicates approval of the first CFTR-modulating therapy (ivacaftor). Box colours indicate applicability in the sense of CF specifity and quality of validation [5,23,26,27,28,29,30,31,32,33,34,35,36,37,39,40,41,42,43,44,45,46,47,48,49,50,51,52,53,54,55,56,57,58,59,60,61,62,63].

Prior to the approval of the first CFTR-modulating therapy ivacaftor by the FDA in January 2012, 12 out of the 39 articles reviewed herein investigated AS in pwCF (see Table 1 and Figure 3).

Already in 1969, Gracey et al. assessed the effects of *n*-acetyl cysteine on *n* = 5 pediatric pwCF complaining about abdominal pain (AP) **registering the occurrence of AP and palpable abdominal masses** before and after therapy. The authors reported relief of AP in all of *n* = 5/5 pwCF after one week of therapy and a reduction of palpable abdominal masses in *n* = 2/5 pwCF [26].

More than two decades later, in a double-blind crossover study published in 1992, Elliot et al. aimed to assess differences in the effects of pancreatic enzyme replacement therapy (PERT) with “Creon” and “Pancrease” in *n* = 27 children with CF using a **symptom diary** including **AP** and **stool frequency**. The authors did not find significant differences between the groups in regard to AP. However, “Creon” intake was associated with lower stool frequency [27].

**Hospital records** regarding **pain episodes requiring medical intervention and their location** were retrospectively assessed by Ravilly et al. (1996) including a posthumous survey group of pwCF above the age of 5 years (*n* = 55 patients deceased between 1984 and 1993) and a consultation group of pwCF (*n* = 23). Most frequently, chest pain and headache were found, followed by AP in both groups [28].

A **questionnaire** focusing on **gastroesophageal reflux symptoms and their relieving factors** was applied by Ledson et al. (1998), assessing the prevalence of upper GI symptoms in *n* = 50 adult pwCF. In addition, *n* = 10/50 pwCF underwent esophageal manometry and 24 h outpatient pH monitoring. 94% of pwCF (47/50) revealed to have symptoms related to the upper GI tract, and 80% (40/50) had heartburn. Of the *n* = 10 pwCF undergoing esophageal manometry, elevated DeMeester scores were found in *n* = 8/10 which included reflux episodes and pH values [29].

A self-report questionnaire assessing five characteristics of pain was applied by Koh et al. (2005) in *n* = 46 children with CF using Likert-type scales, the validated Face Pain Scale (FPS), validated body outline regions, and a visual analogue scale. The authors aimed to capture the occurrence of acute and chronic pain, more specifically its frequency, intensity, duration, and location, its associated emotional upset, as well as the relationship between pain and disease severity. Participants also reported on their pain management strategies. Primary locations of pain were the abdominal/pelvic region (50%). The majority of children reported that rest, medication, relaxation, heat or cold, family/friends, and distracting activities provided some pain relief. Children with chest pain were found to have significantly lower FEV1% compared to children without chest pain [30].

Baker et al. (2005) hypothesized that PERT could have a positive effect on AS including AP, constipation, gassiness, and daily stool frequencies (diarrhea). In this study, a cohort of *n* = 1215 pwCF of all ages attended at33 Cystic Fibrosis Foundation accredited sites were assessed using a **questionnaire** primarily enquiring about PERT use in order to discriminate exocrine pancreatic insufficient from pancreatic sufficient (PS) pwCF. Also, fecal elastase-1 levels were quantified, defining the EPI threshold as ≤200 μg/g of stool. If EPI was present, the respondent was asked about the **frequency of stomachaches, constipation, and stools**. The authors did not provide information on how data in children with CF aged < 6 years were obtained or whether they used a proxy–parent questionnaire. A high prevalence of GI symptoms including gassiness and constipation was reported, being more frequent in patients with EPI than with PS. However, in patients with EPI, no association between higher PERT dosages and reduced burden of GI symptoms was observed [31].

A **6-month symptom diary** also assessing methods to relieve AP in pwCF was administered to *n* = 9 adults with CF by Obideen et al. (2006). The authors aimed to examine the effect of nocturnal hydration, resulting from drinking plenty of water during the night, on recurrent abdominal pain and recurrent pancreatitis. Beginning 3 months before the initiation of nocturnal hydration and ending 3 months after the initiation of the intervention, patients self-recorded the **frequency and severity of AP**, together with pain medication doses and the volume of water consumed. The authors reported that the severity and frequency of AP decreased after nocturnal hydration implementation [32].

Psychometric properties of the **Memorial Symptom Assessment Scale (MSAS)** [38] were investigated with *n* = 303 adult pwCF by Sawicki et al. (2008). In this cross-sectional study, factor analysis led to the development of 3 subscales (MSAS-PHYS, MSAS-PSYCH, MSAS-GDI) with adequate internal consistency (reliability) and moderate correlation with the Cystic Fibrosis Questionnaire (CFQ-R) and the Cystic Fibrosis Quality of Life (CFQoL). Most prevalent symptoms reported in pwCF were cough (94%), shortness of breath (77%), and lack of energy (77%). Also, GI symptoms were captured including vomiting, change in taste, weight loss, and nausea, but the authors reported that the prevalence of these symptoms was much lower than psychological and respiratory symptoms. Females were observed to have a higher burden of symptoms including cough, nausea, and bloating [33].

A **self-administered questionnaire** about the general **prevalence of pain** symptoms and their characteristics, as well as their treatment and impact on QOL, was applied by Sermet-Gaudelus et al. (2009). *n* = 73 children and *n* = 110 adults with CF completed the questionnaire including **Likert-type** rating scale and **visual analogue scale** recalling the previous month. Overall, 59% of the children and 89% of the adults reported at least one episode of pain occurring during the previous month, and 50% of the children and 70% of the adults reported that pain significantly altered their QOL. In children, the pain was reported most frequently in the abdomen, whereas among adults, the back, head, chest, and also the abdomen were quoted as common sites. AP was mostly treated with antispasmodic medications and PERT augmentation. The results were reproducible with three-month follow-up questionnaires from *n* = 33 pwCF [34].

**Questionnaires** including **open-ended and close-ended questions** were mailed to participants by Stenekes et al. (2009) to gather information on self-reported assessment and self-management of **pain**. A total of *n* = 123 surveyed pwCF responded (response rate of 64%), mentioning their self-management methods to relieve their symptoms, of which *n* = 61/123 (50%) reported abdominal pain. Of those, 44% (*n* = 27/61) used one or more pharmacological treatments to manage AP [35].

**Participants were asked** to provide information about **stool frequency**, **mean stool consistency** (hard, formed, soft, and watery), **mean flatulence** (none, mild, moderate, and severe), and **mean abdominal pain** (none, mild, moderate, and severe) in a multicenter, randomized, double-blind, placebo-controlled study by Graff et al. (2010). Over five days, *n* = 16 children with CF received either PERT (pancrealipase delayed-release 12,000-lipase unit capsules) or identical placebo capsules. Differences in the coefficients of fat absorption and nitrogen absorption were calculated by the investigators by analyzing stool samples and clinical global impression of disease symptoms were rated by the investigator. Not surprisingly, all stool characteristics improved significantly, and stool frequency significantly decreased with pancrealipase, compared to placebo. Simultaneously, symptoms of EPI including abdominal pain, flatulence, and stool consistency were less severe during PERT administration, compared with placebo. Mean stool fat, stool nitrogen, and stool weight were significantly lower during pancrealipase treatment compared to placebo, whereas daily fat and nitrogen appeared to be similar in both therapy arms of the study setup. Furthermore, the authors reported a significantly lower mean stool frequency with pancrealipase along with improvement in other stool and symptom measures. Investigator-assessed clinical global impression indicated worsening with placebo and no significant change with pancrealipase [36].

Using a **28-day symptom diary**, Munck et al. (2011) investigated the **prevalence of recurrent AP** (recurrent abdominal pain, according to Apley’s criteria, i.e., at least three AP episodes severe enough to affect daily activities over a period of not less than 3 months, with attacks in the year preceding the examination in *n* = 130 children with CF aged 8–18 years. Furthermore, methods to relieve recurrent abdominal pain, as well as correlated anxiety levels and quality of life were evaluated. The **Faces Pain Scale-Revised** and five additional questionnaires were administered at two time points, i.e., **Eland Pain location**, pain intensity measured by the **Faces Pain Scale-Revised**, **Mc Gill emotional status**, **Revised Children’s Manifest Anxiety Scale,** and health-related quality of life (**CF-QOL**). As a result, the prevalence of recurrent abdominal pain was only 6% (8/130) of the included pwCF, as per Apley’s criteria. Pain symptoms were most frequently located in the epigastric region and in the right or left iliac fossa. *n* = 5/8 patients had severe pain intensity at the initial visit and in *n* = 6/10 AP lasted 30 min or more [37].

### 3.2. Studies Published after the Approval of the First CFTR-Modulating Therapy

According to our search results for this structured review, 27 studies regarding gastrointestinal symptoms in pwCF recorded with a PROM or a questionnaire were published from the approval of the first HEMT, ivacaftor in January 2012 until 20 November 2022 (see Table 2).

**Table 2:** Studies assessing abdominal symptoms (AS) or abdominal pain (AP) in the era of CFTR modulators.

First Author (Year of Publication)	Study Population *n* = Enrolled Patients (Range/Mean/Median Age)	CF-Specific Questionnaire?	Methods Applied (Questionnaire/Diary/PROM) or Symptoms Assessed	Primary Application of the Questions/Questionnaire Regarding:	Measurement Property (Non-CF/ CF-Specific)	Related to-or Addressing CFTR Modulator Therapy	Main/Primary Finding(s) in Relation to Abdominal Symptoms (AS) or Specifically Abdominal Pain (AP)
Fraquelli (2016) [39]	*n* = 70 pwCF (median age: 13.5 y) *n* = 45 HC	no	not provided ^1^	Relation of AS to abdominal ultrasound	*n*/A	no	Bowel ultrasound abnormalities associated with AS. More ultrasound abnormalities in pwCF
Lechtzin (2016) [40]	*n* = 73 pwCF (age 12–20 y)	no	Online survey on prevalence of pain	Unspecific pain	*n*/A	no	Most prevalent pain location: the abdomen (42%)
Zeybel (2017) [41]	*n* = 12 pwCF (age 17–38 y)	no	Hull Airway Reflux Questionnaire (HARQ) and Reflux Severity Index (RSI)	Effects of IVA on Extra-esophageal reflux (EOR) symptoms	Sensitivity to change	IVA	Significant decrease in mean HARQ and RSI scores. Ivacaftor has beneficial effects on EOR, additional to PPI therapy
Gelfond (2017) [42]	*n* = 10 pwCF with G551D mutation (age 25–50 y)	yes	GI subscale of the CF Questionnaire-Revised (CFQ-R)	Effects of IVA on AS	Sensitivity to change	IVA	7/10 pwCF reported abdominal pain at baseline-no changes during treatment with IVA
Tabori (2017a) [5]	*n* = 131 pwCF (all ages)	yes	JenAbdomen-CF (CFAbd-Score 1.0)	First CF-specific GI-PROM	Content & Construct validity	yes	PI and history of GI surgery is associated with higher burden of AS
Johnson (2017 ) [43]	*n* = 61 PEI patients: (age ≥ 12 y)	no	Pancreatic Exocrine Insufficiency (PEI) Questionnaire (PEI-Q)	Psychometric validation	Content validity	no	Development of a PEI-specific PRO instrument including 36 pwCF and 25 people with chronic pancreatitis (non-CF)
Tabori (2017b) [44]	*n* = 114 pwCFs (all ages)	yes	CFAbd-Score 2.0	Relation of AS to abdominal ultrasound	Content and Construct validity	yes	Pancreatic lipomatosis is associated with a higher burden of AS (*p* = 0.036)
Van Biervliet (2018) [45]	*n* = 25 of 31 pediatric pwCF (age 6.9–12.2 y)	no	Symptom diary on AP and stool frequency and consistency	Effects of probiotics on AS, Microbiome and FC	*n*/A	no	Normalization of gut permeability in 13% after probiotic treatment. Correlation gut permeability and AP. No correlation between FC and AP
Johnson (2019) [46]	*n* = 162 PEI patients: *n* = 71 pwCF + *n* = 91 CP (non-CF) (age ≥ 12 y) & *n* = 60 HC	no	Pancreatic Exocrine Insufficiency Questionnaire (PEI-Q)	Psychometric validation	Reliability and Construct and Content Validity	no	PEI-Q: good internal consistency + test–retest reliability in pancreatic insufficiency (partly CF). PEI-Q demonstrated convergent validity (partly CF) and discriminative ability
Jaudszus (2019) [47]	*n* = 116 pwCF (age ≥ 6 y) *n*= 88 HC	yes	CFAbd-Score 3.0	Psychometric validation	Reliability and Validity	no	Internal consistency: good to excellent Test–retest reliability: good to excellent
Hayee (2019) [48]	*n* = 107 pwCF (mean age 27.8 ± 9.6 y)	no	IBS-SSS, GSRS, CFQ-R and PHQ-9, GAD-7	Abdominal symptoms	Validity	no	47/107 (44%) pwCF reported significant symptoms on the IBS-SSS Score GI symptoms in pwCF captured by GSRS
Papantoni (2019) [49]	*n* = 64 parents of pediatric pwCF (age 2–12 y)	no	Children’s Eating Behaviour Questionnaire (CEBQ)	Appetitive disorders	Reliability	no	Internal consistency of the CEBQ was good. CEBQ can be used to identify appetitive disorders in children with CF
Boon (2019) [50]	*n* = 200/248 pwCF (2- < 18 y) and their parents and HC ^2^	no	PedsQL-GI,-CF CFQ-R, VAS	Psychometric properties of the PedsQL-GI	Reliability and construct validity	no	The PedsQL-GI was shown to be feasible in pwCF and their parents
Boon (2020) [51]	*n* = 148/171 pwCF (age 2- < 18 y)	no	MyCyFAPP containing CF-PedsQL-GI CFQ-R, VAS, and CFAbd-Score 3.0	Effects of MyCyFAPP on GI symptoms	Sensitivity to change	no	Use of the MyCyFApp for PERT dosage leads to symptom reduction assessed with the CF-Peds-QL-GI and CFAbd-Score 3.0 after 6 months
Smith (2020) [52]	*n* = 276 respondents: *n* = 90 pwCF + *n* = 79 family + *n* = 107 HCPs	no	Electronic questionnaire	Effects of GI symptoms on QOL	*n*/A	no	Symptoms mostly affecting QOL in pwCF: stomach cramps/pain, bloating and combined symptoms. Three most pronounced symptoms reported by HCPs: reduced appetite, bloating and constipation
Beaufils (2020) [53]	*n* = 37 pediatric pwCF (age 4- < 18 y)	no	GI-Symptoms Scales 3.0-PedsQL and QOL-Pediatric Inventory 4.0-PedsQL	Relation of AS and intestinal inflammation, FC	*n*/A	no	FC levels correlated with higher burden of GI symptoms and impaired QOL scores
Ng (2021) [23]	*n* = 12 pwCF *n* = 12 HC (age >12–40)	yes	CFAbd-Score 3.0 and PAC-SYM	Oro-caecal transit time in MRI (related to AS)	*n*/A	no	CFAbd-Score tended to be higher in pwCF. No difference in median PAC-SYM scores
Freeman (2021) [54]	*n* = 77 responses 66% pwCF > 16 y, 34% family/caregivers *n* = 590 providers ^3^	no	PAGI-SYM, PAC-SYM, PAC-QOL versus PROMIS GI	PROM feasibility	*n*/A	no	PAGI-SYM, PAC-SYM and PAC-QOL best represent GI symptoms in pwCF according to Community Voice members. BSS + 3 questions added
Dziekiewicz (2021) [55]	*n* = 22 pediatric pwCF (age 4–18 y) *n* = 10 placebo pwCF	no	not provided ^4^	Effects of PPI on AP and GERD- symptoms	*n*/A	no	Significant reduction in the severity of AP and GERD symptoms in both groups: treated with omeprazole or placebo
Sathe (2021) [56]	*n* = 402 pwCF (aged ≥ 2 y)	no	PAGI-SYM, PAC-SYM, PAC-QOL + SSQ	Electronic PROM feasibility	Test–retest reliability	no	PROM administration seems feasible in an electronic setting; adequate response rates
Raun (2022) [57]	*n* = 18 pediatric pwCF with PI (age 6–17 y)	yes	CFAbd-Score 2.0	PERT timing (before/after meals)	*n*/A	no	No difference in AS regarding time of PERT administration (prior or after meals). No correlation between FC and AS (small sample size)
Jaudszus (2022) [58]	*n* = 41 pwCF & *n* = 27 HC (all ages)	yes	CFAbd-Score 3.0	AS and intestinal inflammation	Construct validity	no	FC levels were significantly higher in pwCF reporting relevant abdominal pain
Roda (2022) [59]	*n* = 23 pediatric pwCF (age < 18 y)	no	not provided ^5^	AS and intestinal inflammation	*n*/A	no	No correlation between FC and AS
Mainz (2022) [60]	*n* = total 107 pwCF *n* = 68 ≥12 y(GER) *n* = 39 ≥18 y(UK) *n* = 45 HC	yes	CFAbd-Score 3.0	Effects of ETI on abdominal symptoms	Sensitivity to change	ETI	Significant and clinically relevant AS reduction during ETI (total CFAbd-Score declined by 29% and its 5 domains by 23–67%). High ability to detect changes fulfilled
Shakir (2022) [61]	*n* = 32 pwCF (median: 32.5 y)	no	HARQ, RSI, SNOT-20, CFQ-R	(Extra-) esophageal reflux and sino-nasal symptoms	Sensitivity to change	ETI	Improvement in all scores at 3 months and sustained at 6 months of starting ETI
Moshiree (2022) [62]	*n* = 402 pwCF (aged ≥ 2 y)	no	PAGI-SYM, PAC-SYM, PAC-QOL + SSQ	Prevalence, severity and impact on QOL of AS	*n*/A	no	PAC-SYM, PAGI-SYM, PAC-QOL total scores higher in ≥18 y group and in female pwCF
Schwarzen-berg (2022) [63]	*n* = 256–267 of 438 pwCF ^6^ (aged ≥ 12 y)	no	PAGI-SYM, PAC-SYM, PAC-QOL + SSQ	Effects of ETI on GI symptoms	Sensitivity to change	ETI	Statistically significant but clinically irrelevant changes in GI symptoms after 6 months of ETI

^1^ assessed seven GI symptoms. ^2^ details about number of HC were published elsewhere. ^3^ medical providers, dietitians, and nurses. ^4^ symptoms assessed were: cough intensity, AP and GERD symptoms. ^5^ symptoms assessed were: AP, constipation and diarrhea. ^6^ *n* = 263/438, *n* = 267/438 and *n* = 256/438 completed the PAGI-SYM, PAC-SYM and PAC-QOL, respectively. AS: abdominal symptoms; BSS: Bristol Stool scale; CEBQ: Child Eating Behaviour Questionnaire; CF: Cystic Fibrosis; CF-PedsQL-GI: Pediatric Quality of Life Inventory GI Symptoms (PedsQL-GI) Module; CFQ-R Cystic Fibrosis Questionnaire-Revised; CP: Chronic Pancreatitis; EOR: Extra-esophageal Reflux; ETI: Elexacaftor/Tezacaftor/Ivacaftor; FC: Fecal Calprotectin; GAD-7: Generalized Anxiety Disorder 7; GER: Germany; GERD: Gastroesophageal Reflux Disease; GI: gastro-intestinal; GSRS: Gastrointestinal Symptom Rating Scale; HARQ: Hull Airways Reflux Questionnaire; HC: Healthy Controls; HCP: Health Care Professionals; IBS-SSS: Irritable bowel syndrome (IBS)–Symptom Severity Score; IVA: Ivacaftor; MRI: Magnetic Resonance Imaging; MyCyFAPP: My Cystic Fibrosis Application; *n*/A: not applicable; PAC-QOL: Patient Assessment of Constipation- Quality of Life; PAC-SYM: Patient Assessment of Constipation-Symptom Severity Index; PAGI-SYM: Patient Assessment of upper Gastrointestinal Disorders-Symptom Severity Index; PedsQL-GI: Pediatric Quality of Life Inventory; PEI: Pancreatic Exocrine Insufficiency; PEI-Q: Pancreatic Exocrine Insufficiency Questionnaire; PERT: Pancreatic Enzyme Replacement Therapy; PHQ-9: Patient Health Questionnaire; PPI: Proton-Pump Inhibitor; PROM: Patient Reported Outcome Measure; PROMIS-GI: Patient-Reported Outcomes Measurement Information System; pwCF: people with cystic fibrosis; QOL: Quality of Life; RSI: Reflux Symptom Index; SNOT-20: Sinonasal Symptom Test 20; SSQ: Stool-Specific Questionnaire (adapted from Bristol Stool-Scale + 3 stool consistency related questions); UK: United Kingdom; VAS: Visual Analogue Scala; y: year.

#### 3.2.1. Studies Not Focused on Assessing Effects of HEMT

In 2016, Fraquelli et al. studied the correlation between **GI symptoms and bowel ultrasound abnormalities** in 70 pwCF and 45 healthy controls, identifying significantly more ultrasound abnormalities in the cohort of pwCF. Enlarged lymph nodes (64%), bowel loop dilatation (55%), and thick corpuscular intraluminal content (49%) were the most frequent findings in the cohort of pwCF, and at least one of the seven GI symptoms reported therein (AP, diarrhea, bloating, constipation, nausea, vomiting, and GERD) was prevalent in 60% of this cohort. Furthermore, compared to pwCF without pain, pwCF complaining about recurrent AP displayed significantly more ultrasound abnormalities, including bowel wall hypervascularization and evidence of bowel loop intussusception. Further details about the methods and tools applied to record AS were not provided [39].

In the same year, Lechtzin et al. (2016) reported on 73 pediatric pwCF completing an **online survey** to describe the **prevalence, severity, and location of pain** [40]. The online questionnaire contained 188 items including 15 pain-related questions adapted from a questionnaire previously used by Palermo et al. (2006) [64] and the **Brief Pain Inventory** [65]. Additionally, the **CFQ-R** and the **Pain Catastrophizing Scale** [66] were administered to the study participants. At baseline, 89% of the patients reported a prevalence of pain other than “everyday kinds of pain” (as used in the Brief Pain Inventory) within the past three months. In 80% of the participants with pain prevalence, pain was short-lived with mild to moderate severity, according to previous definitions of pain duration and severity [67,68]. According to the study, pain occurred relatively infrequently, as 43% of the participants reported experiencing pain less frequently than once a month, 23% one to three times per month, and 35% once a week or more. The abdomen was the location identified as the most troublesome (42%) [40].

Beaufils et al. (2020) categorized a cohort of *n* = 37 children with CF into a “low FC group” defined as fecal calprotectin (FC) levels ≤ 250 μg/g, and a “high FC group” with a cutoff above 250 μg/g. Participants in the high FC group (*n* = 5) had a significantly higher burden of GI symptoms and worse QOL scores assessed by the **Gastrointestinal Symptoms Scales 3.0-PedsQL**^TM^, which includes 74 questions grouped in 14 GI-related subscales, and the **Quality of Life Pediatric Inventory 4.0-PedsQL**^TM^, respectively. Gastrointestinal Symptoms Scales 3.0-PedsQL^TM^ scored higher with GI symptoms in the “high FC group”, compared to the “low FC group”: “Total”, “Heart Burn and Reflux”, as well as “Nausea and Vomiting” by children and their parents as well as “Gas and Bloating” by children and “Stomach Discomfort When Eating” by their parent [53].

In a double-blind placebo crossover multicenter trial, Van Biervliet et al. (2018) evaluated the clinical effects of probiotics (*Lactobacillus Rhamnosus SP1* and *Bifidobacterium animalis* spp. *BLC1*) on several clinical parameters, including an **abdominal symptom diary**, FC, the intestinal microbiome, and gut permeability assessing the lactulose/mannitol ratio. Twenty-five of the 31 enrolled children with CF (median age: 9.3 years (6.9–12.2)) finished the study. In regard to the abdominal symptom diary, the only information provided is that patients were requested to daily record stool frequency and consistency, abdominal pain, as well as all treatment changes. The authors reported normalization of gut permeability in 13% of participants after probiotic treatment and a correlation between gut permeability results and abdominal pain. Interestingly, neither a correlation of FC and abdominal pain nor a shift at the Phylum level was found after the probiotic supplementation [45].

Hayee et al. (2019) utilized tools validated for other non-CF pathologies, including the **GI Symptom Rating Scale (GSRS)** (11 upper- and lower-GI symptoms) and the **Irritable Bowel Syndrome Symptom Severity Score (IBS-SSS)** (five items: abdominal pain intensity, abdominal pain frequency, abdominal distension, dissatisfaction with bowel habits, and interference of symptoms on life), and the CF-specific **Cystic Fibrosis Questionnaire (CFQ-R)** to determine the prevalence of chronic GI-related symptoms in *n* = 107 pwCF. They found that 44% of pwCF had significant symptoms on the IBS-SSS (score > 75) and that 65% of the included pwCF fulfilled the ROME IV criteria for IBS [69]. GI symptoms, assessed with the GSRS affecting the lower GI tract, were mostly reported as moderate to severe. No differences were seen in IBS-SSS and GSRS scores from patients calculating their PERT dosage and those only estimating the required dose. Furthermore, no correlation was found between the CFQ-R (even the digestion domain) and IBS-SSS or GSRS [48].

Papantoni et al. (2019) administered a brief parent report **Child Eating Behavior Questionnaire (CEBQ)** to *n* = 64 parents of children with CF aged 2–12 years. Psychometric properties such as internal consistency were good, according to Cronbach’s alpha. According to the study, the sub-scale “Slowness in Eating”, one of the four sub-scales assessing “food avoidance”, correlated negatively with BMI z-scores. Conversely, the sub-scales “Food Responsiveness”, “Enjoyment of Food”, and “Emotional Overeating”, 3 of the 4 sub-scales assessing “Food approach”, correlated positively with BMI z-scores. The authors concluded that the application of the CEBQ can be effectively used to identify children with CF with appetitive disorders associated with difficulty in weight gain and could be useful in developing therapeutic interventions [49].

In an **electronic questionnaire** (SurveyMonkey) regarding GI symptoms targeting pwCF, parents, and healthcare professionals, Smith et al. (2020) inquired about the effects of GI symptoms on QOL in pwCF. The online survey was designed by the CF community (pwCF, their family members, and health care professionals), including open and closed questions as well as optional free-text fields, which were evaluated qualitatively and quantitatively, e.g., by using Likert-scales. The survey was promoted via web-based platforms including @questionCF on Twitter. PwCF reported wind/gas, bloating, and stomach cramps as the symptoms mostly affecting their QOL. Unlike pwCF, healthcare professionals quoted reduced appetite, bloating, and constipation as their top 3 symptoms impairing QOL. Almost all healthcare professionals (94%) thought medications would help to relieve GI symptoms, but only 58% of lay respondents agreed. health care professionals suggested that laxatives and antacid medication helped the most, whereas, additionally to antacid medication, pwCF reported that pancreatic enzyme supplementation had the best effects to relieve their symptoms [52].

Johnson et al. published two consecutive studies in 2017 and 2019 reporting on the development and validation of the **Pancreatic Exocrine Insufficiency Questionnaire (PEI-Q)**. This PEI-specific instrument focused on patients with chronic pancreatitis or CF. The first qualitative research in 61 patients with PEI (36 pwCF and 25 people with chronic pancreatitis) and 10 physicians generated a conceptual model with 6 symptom concepts and 6 impact domains, which led to the development of a preliminary version of a PROM with a total of 45 items. Eighteen items were excluded following cognitive debriefing patient interviews and physicians’ input, resulting in a final PROM with 27 items considered for further validation steps [43].

In a subsequent study from 2019 including 71 pwCF (25.7 ± 10.9 years) and 91 patients with chronic pancreatitis (57.1 ± 12.2 years), Johnson et al. assessed the psychometric properties of the PEI-Q. In both patient groups, chronic pancreatitis and CF, PEI was diagnosed by a physician. Patients were required to have experienced PEI symptoms within the past three months and to have been on EPI treatment for a maximum of six years. Psychometric properties of the PEI-Q were evaluated for internal consistency (Cronbach’s alpha: 0.77–0.82), test–retest reliability (ICC: 0.73–0.87), as well as for convergent validity (correlation between PEI-Q and the Gastrointestinal Quality of Life Index (GIQLI)). Exploratory factor analysis on the revised 18-item PEI-Q revealed three domains: “abdominal symptoms” (7 items: stomach pain (*n* = 1), bloating (*n* = 1), stomach noises (*n* = 1), passing gas (*n* = 2), nausea (*n* = 1) and appetite (*n* = 1)), “bowel movements symptoms” (6 items: diarrhea (*n* = 1), stool colour (*n* = 1), stool odour (*n* = 1), fat or oil in the stool (*n* = 1) and need to rush to get to a toilet (bowel urgency, *n* = 1) or be close to a toilet (*n* = 1), and “impacts” (5 items assessing the impact on avoiding fatty foods, concentration, embarrassment, worry, and social activities). Known-group validity tests were conducted by comparing *n* = 162 PEI patients and *n* = 60 healthy controls. The PEI-Q was shown to discriminate within PEI symptom severity, as well as between PEI patients and healthy controls. However, information about a PEI diagnostic test was not available/collected in half of the participants [46].

In a randomized, double-blind, placebo-controlled trial over 12 weeks, Dziekiewicz et al. (2021) assessed the effect of proton pump inhibitor therapy (omeprazole) on GERD symptoms in a cohort of *n* = 22 pediatric pwCF, compared to *n* = 10 pwCF receiving placebo. **Patients were asked** to rate **cough intensity**, **AP**, and typical **GERD symptoms**, i.e., heartburn and/or regurgitation, on a **visual analogue scale** (1–10 points) regarding the past 7 days prior to study inclusion. After 12 weeks of treatment, significant reductions in AP severity and typical GERD symptoms were observed. Interestingly, this occurred in both the omeprazole and the placebo group. Reductions in some 24 h pH-monitoring parameters were also observed [55].

Raun (2022) investigated the effects of timing of PERT administration (Creon^TM^/Kreon Für Kinder^TM^) on GI symptoms using the **CFAbd-Score** (version 2.0, see below) in a randomised cross-over design in *n* = 30 pwCF allocated to two subgroups according to their time of PERT ingestion, i.e. either before or after meals for two consecutive periods of four weeks. Of the *n* = 30 participants, *n* = 22 completed the study, but data from participants below 6 years (*n* = 4) were excluded, reducing the sample size to *n* = 18. The authors did not find significant changes in GI-related abdominal pain, bowel habits, or GI-related QOL when participants switched PERT supplementation from before to after meals and/or vice versa. The hypothesis that PERT administration after meals could be beneficial regarding AS, which had been proposed in some previous studies, did not attain significance in this study [57].

In a prospective study including *n* = 23 pwCF aged < 18 years, Roda et al. (2022) analyzed the association between FC and AS, as well as FC and histological inflammatory features of rectal mucosa. **Patients were asked to report the occurrence of constipation, AP, and diarrhea** during the two weeks preceding FC measurement. Rectal mucosa specimens were obtained from *n* = 11/23 pwCF. There was a correlation between elevated FC levels and the presence of inflammation in rectal biopsies. However, the GI symptoms enquired in this small study were not associated with elevated FC levels [59].

In 2019, as part of the European multicentric HORIZON 2020 project, the electronic application “MyCyFAPP” was designed to optimize and personalize PERT dosages for pwCF. Within this context, Boon et al. (2019) aimed to investigate the psychometric characteristics of the **Pediatric Quality of Life Inventory GI (PedsQL-GI)** in a cohort of *n* = 248 children with CF (age: 2 to <18 years) and their parents. This generic symptom measurement instrument comprises 74 questions in 14 GI-related subscales, and it has been validated in patients with gastrointestinal diseases such as Crohn’s disease, gastroesophageal reflux disease, and functional gastro-intestinal disorders [70]. In the international European MyCyFAPP project, *n* = 200/248 PedsQL-GI questionnaires were completed at baseline. Data from a group of healthy controls from the United States (US) that had been previously published elsewhere was included. The aim of this prospective multicenter study was to evaluate the reliability of the PedsQL-GI and its relation with CFQ-R and the visual analogue scale at baseline (M0) and after 3 months (M3). PedsQL-GI scores correlated positively with BMI z-scores and age, and there was no association with the total PERT dose. Measures of internal consistency for the PedsQL-GI and most of its subscales were high. The intraclass correlation coefficient between M0 and M3 was moderate to good in most of the sub-scales of the PedsQL-GI, indicating an excellent test–retest reliability. A weak but significant correlation was observed between the PedsQL-GI, most of the CFQ-R subscales, and the visual analogue scale. Positive associations were found between PedsQL-GI and anthropometric parameters like age and BMI z-scores. No correlation was found between the PedsQL-GI and total PERT dose. Conclusively, due to its reliability and construct validity, the PedsQL-GI proved to be feasible for pwCF and their parents. Based on that, a shortened version consisting of 54 questions belonging to 8 subdomains was developed, and renamed CF-Ped sQL-GI [50].

In a subsequent study, Boon et al. (2020) assessed the impact of “MyCyFAPP” on GI symptoms, QOL, general well-being, and nutritional status in pwCF. Data from *n* = 148/171 pwCF (median age: 8.5 years (5.5, 12.85)) were prospectively collected at baseline, at 3 months, and after 6 months with the CF-PedsQL-GI. As a secondary GI-PROM, the **CFAbd-Score** (version 3.0, see below) was implemented in the study. The resulting scores obtained with both questionnaires showed significant improvement in GI symptoms after 6 months of using the MyCyFAPP in pwCF and their parents [51].

In 2020, Ng et al. used the **CFAbd-Score 3.0** and **PAC-SYM** to assess GI symptoms in *n*= 12 pwCF over the 2 weeks preceding magnetic resonance imaging analysis of oro-caecal transit times, colonic and gastric volumes, as well as intestinal increased small bowel water content. Median CFAbd-Score and PAC-SYM were higher in pwCF compared to the control group, without attaining significance and with a markedly greater difference obtained using the CFAbd-Score. Longer oro-caecal transit times and larger colonic volumes were observed in the CF group. However, as the increased small bowel water content was concomitantly higher, the authors suggested an impaired emptying function in the small bowel, but not in the stomach. These findings did not correlate with GI symptoms, possibly due to the small sample size, according to the authors [23].

#### 3.2.2. Studies Focused on Assessing Effects of HEMT Therapies

Regarding studies addressing the effects of CFTR modulator therapies on GI symptoms, the prospective study by Zeybel et al. (2017) explored the relation between therapy with ivacaftor (IVA) and extra-esophageal reflux symptoms in *n* = 12 pwCF [71]. Data were collected at baseline, as well as 6, 26, and 52 weeks after commencing therapy with ivacaftor. The study implemented two questionnaires developed to assess extra-esophageal reflux symptoms which are validated for diseases other than CF: the **Hull Airway Reflux Questionnaire (HARQ)**, designed to assess chronic cough and hypersensitivity syndrome [72], and the **Reflux Symptom Index (RSI)**, initially validated to evaluate laryngopharyngeal symptoms secondary to reflux [73]. It comprises the following 9 items: “Hoarseness or a problem with your voice”, “Clearing your throat”, “Excess throat mucus or postnasal drip”, “Difficulty swallowing food, liquids, or pills”, “Coughing after you ate or after lying down”, “Breathing difficulties or choking episodes”, “Troublesome or annoying cough”, “Sensations of something sticking in your throat or a lump in your throat”, and “Heartburn, chest pain, indigestion, or stomach acid coming up”. Nevertheless, during therapy with ivacaftor, both scores declined at all three time points, and even a decrease in symptom severity in pwCF was observed during proton pump inhibitor medication.

Also focusing on the effects of a CFTR-modulating therapy, Gelfond et al. (2017) assessed the intestinal transit time in *n* = 10 pwCF carrying a gating mutation using a wireless motility capsule before and during therapy with ivacaftor. Self-reported AP was recorded during both time frames, i.e., before and during ivacaftor therapy, using the **GI subscale of the CF Questionnaire-Revised (CFQ-R subscale)**, which includes questions on “problems with wind”, “diarrhea”, and “abdominal pain” [74,75]. However, despite a significant increase in mean pH values between 8 and 24 min after gastric emptying, suggesting increased bicarbonate secretion, no changes in abdominal pain or regional intestinal motility were observed [42].

In a prospective observational study including *n* = 32 pwCF with advanced lung disease, Shakir et al. (2022) investigated the longitudinal effects of elexacaftor–tezacaftor–ivacaftor (ETI) on esophageal, extra-esophageal laryngopharyngeal and sinonasal symptoms in pwCF. Patients starting ETI completed the **Reflux Symptom Index (RSI)**, the **Hull Airways Reflux Questionnaire (HARQ)**, the **sinonasal symptom test 20 (SNOT-20)** [76], and the **CF questionnaire revised (CFQ-R)** at baseline, and at 3 and 6 months after initiating ETI. After 3 months of therapy with ETI, decreases in RSI, HARQ, and SNOT-20 scores exceeded the minimal clinically significant change of 5, 16, and 8 points, respectively. Gastro-esophageal reflux, extra-esophageal reflux, and sinonasal symptoms improved in all scores at 3 months of starting ETI and sustained at 6 months of starting ETI [61].

The **Patient Assessment of Constipation-Symptom Severity Index (PAC-SYM)** [77], the **Patient Assessment of Upper Gastrointestinal disorders-Symptom Severity Index (PAGI-SYM)** [78], and the **Patient Assessment of Constipation-Quality of Life (PAC-QOL)** [79] have been implemented in multicenter studies in the US referred to as GALAXY and PROMISE.

Initially, Freeman et al. (2021) surveyed members of “Community Voice”, including pwCF older than 16 years and caregivers, to select items for PROMs that “best described their overall GI health or the GI health of the person they care for, as well as the ease, feasibility and missing questions for each survey” [54]. Subjects were asked to complete two sets of questionnaires: the Patient-Reported Outcomes Measurement Information System (PROMIS) GI symptom scale and a combination of three other PROMs, i.e., the PAC-SYM, PAGI-SYM, and PAC-QOL. As an example, the PAC-SYM, previously developed and validated in non-CF patients suffering from constipation as a lower GI disorder, contains 12 items assigned to 3 subscales: stool symptoms, rectal symptoms, and abdominal symptoms. In these patients, this PROM had been found to have high internal consistency and test–retest reliability [77]. Also, the PAGI-SYM had been designed and validated in non-CF patients suffering from upper GI symptoms. It includes 20 items attributed to the 6 subscales: heartburn/regurgitation, fullness/early satiety, nausea/vomiting, bloating, upper abdominal pain, and lower abdominal pain. In non-CF patients with GERD, dyspepsia, or gastroparesis, the PAGI-SYM revealed good reliability and evidence supporting construct validity [80]

The recall time frames for these PROMs were modified to 1 week, despite the PROMs being developed and validated (for non-CF pathologies) for a recall period of 2 weeks. Based on 77 responses (66% pwCF, 34% family caregivers), PAC-SYM, PAGI-SYM, and PAC-QOL were reported to better represent their or their child’s/family member´s GI health and were easier and more feasible to fill out. Based on the feedback from Community Voice, a **modified Bristol Stool Scale** was added on to assess stool characteristics, as well as three individual questions (stool pattern, frequency, and incontinency) [54].

In a consecutive GALAXY publication, Sathe (2021) assessed the utilization and feasibility of a mobile application implementing the set of questionnaires selected in their previous study (see above). These ePROMs were prospectively administered to *n* = 402 initially enrolled pwCF aged ≥ 2 years. Using a mobile application, participants were asked to subsequently complete the ePROMs at weeks 1, 2, and 4 after the enrollment visit, except for PAC-QOL, which was only administered at enrollment (week 0) and week 4. The authors evaluated recall rates and the score variability over time for PAC-SYM, PAGI-SYM, and PAC-QOL. Overall, the full completion rate for all 4 ePROMs for all follow-up weeks was 77.6%, being higher among those ≥ 18 years of age compared to those < 18 years of age. Low variability in score changes for the questionnaires remained stable from week 0 to each follow-up over the 4 weeks. The authors concluded that the ePROMs seemed feasible to record GI symptoms in pwCF [56].

In a follow-up publication from the GALAXY team, Moshiree et al. (2022) aimed to determine the prevalence, severity, and impact of GI symptoms on QOL of pwCF [62]. As a secondary aim, they compared symptom profiles and QOL in pwCF with and without therapies on proton pump inhibitors, laxatives, PERT, and CFTR modulators. By drawing on the same context as in their previous publication [56], the report regards *n* = 402 pwCF ≥2 years of age who filled out the set of ePROMs on a weekly basis for a month (at weeks 0, 1, 2, and 4). According to this study, proton pump inhibitor use and constipation medications were associated with worse GI symptom scores. The most common symptoms experienced by all age groups were bloating, followed by fullness/early satiety, and lower and upper abdominal pain. High scores for all three ePROMs were observed in patients aged ≥18 and in females. No differences in ePROMs scores were observed between pwCF with and without CFTR modulator therapy [62].

As part of the consecutive PROMISE study, Schwarzenberg et al. (2022) investigated ETI-induced effects on GALAXY ePROMs GI outcomes in pwCF aged ≥12 years who completed, on a mobile application, at least 1 ePROM before and another one during therapy with ETI at 1, 3, and/or 6 months. Faecal samples were simultaneously collected for FC analysis before and 6 months after ETI initiation. PAGI-SYM, PAC-SYM, and PAC-QOL were completed before and during ETI therapy by *n* = 263, *n* = 267, and *n* = 256 pwCF, respectively. Except for PAC-SYM Bloating and PAC-SYM Rectal Symptoms estimated mean changes from baseline to month 6 showed statistically significant lower severity levels in GI symptoms for all domains. However, declines accounting for −0.03–0.25 points did not surpass PAC-SYM’s minimal clinically important difference, which, e.g., for PAC-SYM is estimated to be −0.6 [81]. Consequently, the authors stated that “observed changes are unlikely to be clinically significant over the total population of participants”. In addition, the authors found a highly significant and clinically relevant decline in FC during therapy with ETI, but there was no association between FC changes and changes in the therein-assessed GI symptoms [63].

The first version of the CF-specific **CFAbd-Score (version 1.0)** (initially named JenAbdomen–CF score), developed and validated according to FDA guidelines for the development of a PROM [82], implemented 19 items including a modified Bristol Stool Scale. Items and wording for the PROM were selected and adapted in an iterative process repeatedly including pwCF, their proxies, and CF specialists from different fields, including pulmonologists, gastroenterologists, pediatricians, diabetologists, nutritionists, psychologists, CF-nurses, and others. Applying the PROM for the first time in *n* = 131 pwCF of all ages, Tabori et al. (2017) found an elevated burden of AS in pwCF with a history of CF-related GI manifestations including meconium ileus, DIOS, rectal prolapses, laparotomy, small bowel resection, as well as those with exocrine pancreatic insufficiency and intermittent *Pseudomonas aeruginosa* colonization. In addition, pwCF carrying a G551D mutation (*n* = 17/131) had a lower prevalence of GI symptoms, compared to those without this mutation. Of note, 10 of the 17 pwCF carrying a G551D mutation received ivacaftor therapy at this time point [5].

In the same year, Tabori et al. (2017) correlated findings from abdominal ultrasound, assessing 17 CF-typical pathologies, with the results obtained with the second version of the CF-specific CFAbd-Score (version 2.0) in *n* = 114 pwCF of all ages. The **CFAbd-Score 2.0**, which according to FDA guidelines, iteratively underwent the above-mentioned revisions including pwCF, their proxies, and the multidisciplinary care teams, at that time included 7 additional items on GI-related QOL, resulting in a total of 26 items. Therein, the most important finding was that pwCF with pancreatic lipomatosis in ultrasound had a higher burden of GI symptoms. Specifically, they reported significantly more abdominal pain, flatulence, heartburn, and reflux. Furthermore, pwCF with a microgallbladder had higher pain levels during bowel movements and steatosis hepatis was associated with higher fatty stool frequencies. Furthermore, pancreatic sufficient pwCF displayed lower rates of abnormalities in the Ultrasound-17-Score, which was also associated with lower CFAbd-Scores [44].

Psychometric validation of the CFAbd-Score (version 3.0), which includes two additional questions, was the primary aim of the publication by Jaudszus et al. in 2019. Including *n* = 116 pwCF aged ≥6 years and *n* = 88 healthy controls, the CFAbd-Score was shown to meet essential requirements regarding FDA guidelines for the development of a PROM [83], including content validity, construct validity (known-groups validity), reliability in regard to internal consistency, and test–retest reliability [47]. Furthermore, a significant and clinically relevant difference in total CFAbd-Scores between pwCF and healthy controls was found, accounting for 17.3 versus 8.0 points, respectively.

Again, with the use of the **CFAbd-Score 3.0** in *n* = 41 pwCF and *n* = 27 healthy volunteers’, Jaudszus et al. (2022) assessed the correlation between intestinal inflammation and AS. Abdominal symptoms and impaired GI-related QOL were studied in relation to inflammation markers such as FC, M2-Pyruvate Kinase (M2-PK), interleukins (IL)-1β, IL-6, IL-8, and neutrophilic elastase (NE). FC levels were significantly higher in pwCF reporting relevant AP with the CFAbd-Score. Furthermore, in pwCF with FC levels above 50 μg/g of stool, a significant positive correlation between FC concentrations and the GI-related QOL domain scores was found [58]. 

It is noteworthy that despite not being within the time frame of this review, Caley et al. (2023) recently showed that **CFAbd-Scores 3.0** are markedly higher in pwCF additionally suffering from CF-related diabetes. In detail, their burden of AS quantified as total CFAbd-Scores resulted to be 28% higher in pwCF additionally suffering from CFRD (18.4 versus 25.4 points). These marked differences also resulted in significantly higher scores for domains “GERD” +46% (19.2 versus 35.3 points), “Disorders of appetite” +53% (7.4 versus 15.9 points), and “GI-related QOL” (+42% with diabetes). Differences between the two groups were also marked, albeit not reaching significance, for the “Pain” (+ 21%) and “Bowel movement” domains, which, however, only revealed a +4% difference between both groups [84].

In 2022, Mainz et al. published the first study assessing the impact of the novel triple CFTR modulating therapy with elexacaftor–tezacaftor–ivacaftor (ETI) on GI symptoms captured with the **CFAbd-Score 3.0**, the only CF-specific GI PROM developed and validated following FDA guidelines [60]. Significant and clinically relevant decreases in the total CFAbd-Score (−29%) and its five domains “Pain” (−37%), “GERD” (−48%), “Bowel movement” (−23%), “Disorders of appetite” (−67%), and “GI-related QOL” (−61%) were observed in a cohort of 107 pwCF from Germany (*n* = 68; ≥12 years) and the UK (*n* = 39; ≥18 years) during the first 24 weeks of ETI therapy. In the “GERD” and “Disorders of appetite” domains, pwCF reported a reduction of the burden of symptoms even lower than the level of the included healthy cohort. This study represents a further step in the development and validation of the CF-specific score: the final version of the CFAbd-Score was shown to meet essential criteria in regard to sensitivity to changes during therapies, as recommended by FDA guidelines for the development of a PROM [82].

## 4. Discussion

The number of studies focusing on gastrointestinal symptoms in pwCF as an outcome markedly increased over the recent years. This has been fostered by the substantial improvement in the clinical course and the survival of pwCF with the availability of highly effective CFTR modulator therapies (HEMT) [85,86]. In contrast to most of the previous therapeutic milestones in CF therapy, HEMT does not only change the course of one organ manifestation. Rather, in patients carrying sensitive CFTR mutations, oral intake of CFTR-modulating therapies appears to correct, and potentiate CFTR function in all organs affected by the multi-organ disease. Consequently, unlike previous studies on CFTR modulator therapies, which had pulmonary function as a primary outcome, there is an urgent need for future studies to extend the focus [25,87]. In order to do so, tools for accurate assessment of changes in the multi-organ dysfunction of the digestive system due to CFTR malfunction are urgently required [12,88]. In parallel, also the CF community has clearly addressed the need to focus on the burden of GI symptoms in pwCF. This was evident in two recent surveys from 2018 and 2023 [24,89], in which the “relief of gastrointestinal symptoms such as stomach pain, bloating, and nausea” remained one of the top 10 research priorities in cystic fibrosis, according to the respondents.

As reported in this review, despite the early attempts to identify and record AS reported as early as 1969 [26], many studies addressing outcomes related to AS in pwCF reveal the following trends. Many included single or grouped questions on GI complaints, e.g., addressing pain or constipation, which mostly lacked validation for pwCF [37,45]. Furthermore, the CFQ-R was implemented in the vast majority of large studies performed during the past two decades, which includes 50 items in its adult version. These are implemented in nine QOL domains (physical, role/school, vitality, emotion, social, body image, eating, treatment burden, and health perceptions) and 3 symptom scales (weight, respiratory, and digestion). Whereas the respiratory domain of the CFQ-R has shown to be sensitive to HEMT effects [90], sensitivity to change for the burden of GI symptoms, with regard to this “game changer” in CF therapy has not been reported to our knowledge [42]. We attribute this to the comparatively small number of GI symptom items included in the “GI subscale” of the CFQ-R, namely “problems with “wind”, “diarrhea”, and “abdominal pain” as abdominal complaints, which may be complemented by “eating problems” located outside the “GI subscale” [61,74]. Consequently, to our knowledge, no substantial changes in the GI subscale of the CFQ-R were observed in various studies assessing therapeutic approaches other than HEMT [50,51].

Accordingly, the complex multi-organ abdominal involvement in pwCF requires capturing a broader spectrum of abdominal symptoms. This has prompted GI specialists in different countries to implement GI-PROMs in CF studies, which have been successfully developed and validated for defined GI pathologies [50,51] including inflammatory bowel diseases, upper GI pathologies like GERD, lower GI pathologies like constipation [56,62,63] or the frequent irritable bowel syndrome [48]. In MyCyFApp, the approach taken involved the modification and validation of the PedsQL-GI, shortening the pediatric GI questionnaire, referred to as CF-Peds-QL-GI, and applying it for optimization of PERT dosages while assessing changes in GI symptoms of the included children with CF [51]. The authors found a degree of reduction in abdominal symptoms assessed with the CF-Peds-QL-GI after 6 months. In this study, the CFAbd-Score 3.0 (see below) was also implemented in parallel, revealing improvement in abdominal symptoms after 6 months of intervention in MyCyFApp.

With availability of HEMT for a majority of pwCF carrying a F508del mutation [91,92], sensitive tools to capture effects induced by these therapies needed to be established. HEMT promptly emerged as a game changing intervention, as during its implementation multiple studies following pwCF showed an improvement in pulmonary function and lung clearance index, in upper airway involvement and sweat tests [93,94], as well as regarding the CF-related digestive insufficiency of pwCF. Furthermore, a recent study assessing intestinal current measurement obtained by rectal biopsies has shown positive effects of ETI on CFTR function in the intestinal epitelium of *F508del* heterozygous and heterozygous pwCF [95]. Already with early HEMT therapies with ivacaftor in carriers of a gating mutation, using capsule endoscopy, Gelfond (2017) [42] showed that gastrointestinal hyperacidity significantly and clinically relevantly improves with ivacaftor. Later, Shakir et al. (2022) found significant reductions in GERD in pwCF treated with ETI, using the Hull Airways Reflux Questionnaire (HARQ) and Reflux Symptom Index (RSI) [61]. Above all, overall digestive insufficiency markedly improved in the vast majority of pwCF receiving this therapy, as made evident by stabilization of body weight with HEMT [87,91,92,96]. Furthermore, incidence of pancreatitis markedly declined and some patients carrying gating mutations even regained pancreatic sufficiency. Of note, in pwCF carrying a G551D mutation pancreatic insufficiency appears to manifest later, in the first decade of life without PERT [41,97,98,99].

Recently, after initiation of ETI in a large cohort of pwCF attended in 56 North American CF centers, Schwarzenberg et al. (2022) [63] found a marked and significant decline in fecal calprotectin, the most accepted marker of intestinal inflammation in people with inflammatory bowel syndromes [100]. Nevertheless, in 276 of 402 pwCF followed during ETI implementation in this study, significant declines in the PAC-SYM, PAGI-SYM and PAC-QOL scores did not reach by far the minimal clinically important difference threshold. Adjusted changes ranged between −0.03 and 0.25, clearly below the minimal clinically important difference for, e.g., PAC-SYM, identified to be about −0.60 [56,62,63,81].

In contrast, preliminary results obtained with the CFAbd-Score revealed a significant and highly clinically relevant decline in AS in 107 pwCF during 24 weeks of therapy with ETI [60]. Despite the heterogeneity in the study cohort including pwCF from each 4 CF centers in Germany and the UK, total CFAbd-Scores declined by 29%. Scores for “Pain”, “GERD”, “Bowel movement”, “Disorders of appetite”, and “GI-related QOL” domains decreased by 37%, 48%, 23%, 67%, and 61%, respectively. Recently, these results were confirmed and shown to be sustained over time in 108 pwCF from the Irish/British RECOVER project after 1, 2, 6, and 12 months of therapy with ETI. Declines in total CFAbd -Scores were even more pronounced, with changes of 41% and 35% after 2 and 12 months, respectively. Its five domains also showed significant decreases in a highly clinically relevant range during the mentioned period of time [101].

These results were confirmed in another German cohort followed up with the novel prospective diary version of the PROM, the CFAbd-day2day, cumulatively calculating CFAbd-Scores after 2–4 weeks of therapy with ETI. Nevertheless, notably during the first 2 weeks of the new therapy, the prospective diary revealed within and between subject dynamics of symptoms, even including some transient periods of abdominal symptoms worsening, most pronounced during the first 2 weeks of ETI treatment and mostly stabilizing already in weeks 3–4 of therapy [102].

Accordingly, the CFAbd Score is at present the first and only CF-specific GI-PROM reporting relevant and significant declines in GI symptoms in pwCF from Germany, the UK and Ireland during therapy with HEMT. Simultaneously, these highly consistent findings sustained for up to 12 months show that the CFAbd-Score meets essential criteria with regard to “sensitivity to change”, as recommended in FDA guidelines. We estimate that the 28 items included and grouped in 5 domains, as identified by pwCF, their proxies and CF care takers of different professions in an iterative process as recommended by the FDA, contribute to this PROM’s high sensitivity. Specifically for GI-related studies, we estimate that the 28 items are suitable. Nevertheless, for studies assessing the overall multi-organic effects of new therapies, a reduced number of relevant items may suffice. These items are currently being identified in more than 2000 CFAbd-Scores collected internationally, of which more than 250 Scores were collected before and during a therapy with ETI. The PROM is available in 11 languages as well as in online versions, and, in a digital version, it has recently been integrated into the German CF registry as a diagnostic tool. Furthermore, a diary version of the PROM has been adapted and implemented to prospectively capture GI symptoms during a new therapy with ETI [102] and a pediatric version of the PROM, comprising easy language and pictograms has been developed.

In summary, the need to accurately and reliably explore all manifestations of the multi-organ inherited disease of CF has now generally been well accepted. Therefore, both the respiratory and digestive systems should be regarded when evaluating the clinical course and the burden of symptoms. Whereas questionnaires developed and validated for other GI pathologies underlined the high burden of abdominal symptoms in pwCF, they lacked adequate sensitivity to capture changes induced by HEMT. In contrast, CF-specific GI-PROMs developed and validated according to FDA guidelines proved that therapeutic effects, e.g., induced by HEMT, are associated with a clinically relevant and highly significant reduction in the burden of AS in pwCF.

## Figures and Tables

**Table 1:** Studies assessing unspecific pain and abdominal pain using self-designed questionnaires (no PROMs) and non-CF-specific PROMs before the CFTR modulator therapy era.

First Author (Year of Publication)	Study Population *n* = Included Patients (Range Age)	Methods Applied (Questionnaire/Diary/PROM) or Symptoms Assessed	Primary Application of the Questions/ Questionnaire Regarding:	Main/Primary Finding(s) Regarding Abdominal Symptoms (AS) and/or Abdominal Pain (AP)	Treatment-induced Differences Observed
Gracey (1969) [26]	*n* = 5 pwCF (age 5–19 y)	AP and palpable abd. masses	Effects of *n*-acetylcysteine and AS	Relief of AP in all patients within 1 week. Fecal masses reduced in *n* = 2/5 pwCF	yes
Elliot (1992) [27]	*n* = 27 pwCF (age 5–18 y)	**Symptom diary** on AP and stool frequency	Two types of PERT and AS	No differences observed in regard to AP	no
Ravilly (1996) [28]	*n* = 23 pwCF and *n* = 55 pwCF deceased 1984–1993 (age > 5 y)	**Hospital records** on pain episodes requiring medical intervention	Unspecific pain	Most frequent: headaches and chest pain followed by AP. *n* = 16 patients reported 27 episodes of AP	*n*/A
Ledson (1998) [29]	*n* = 50 pwCF (age 16–50 y)	**Questionnaire** incl. heartburn, regurgitation, and dyspepsia	Upper GI symptoms incl. reflux episodes and pH values (DeMeester Score)	94%pwCF (47/50) reported upper GI symptoms. *n* = 8 of 10 undergoing oesophageal manometry displayed elevated DeMeester Scores	*n*/A
Koh (2005) [30]	*n* = 46 pwCF (age 8.1–17.8 y)	**Self-report questionnaire** incl. LS, FPS, validated body outline regions, and VAS	Unspecific Pain	Most prevalent pain location was the abdomen/pelvis (50%)	*n*/A
Baker (2005) [31]	*n* = 1215 pwCF (all ages)	**Questionnaire** incl. frequency of stomachaches, constipation and stools	Effects of PERT dosages and AP	No decrease in GI symptoms with higher PERT dosages	No
Obideen (2006) [32]	*n* = 9 pwCF (age 45–57 y)	**6-month diary** incl. AP frequency and severity and pain medication	Effect of nocturnal hydration and recurrent pancreatitis/AP	Average of AP severity and frequencies decreased after 3 months of nocturnal hydration	yes
Sawicki (2008) [33]	*n* = 303 pwCF (age 19–64 y)	**MSAS** (non GI-specific HRQOL)	Psychometric properties of MSAS	3 subscales of the MSAS showed good internal consistency (reliability)	*n*/A
Sermet-Gaudelus (2009) [34]	*n* = 73 pwCF (age 1- < 18 y) *n* = 110 pwCF (age 18–52 y)	**Questionnaire** on pain episodes incl. LS and VAS	Unspecific Pain	Pain in children with CF most often in the abdomen, whereas adults, the back, head, chest, and abdomen.	*n*/A
Stenekes (2009) [35]	*n* = 123 pwCF (age 7–60 y)	Questionnaire incl. open-and close-ended questions	Unspecific Pain	50% of participants (61/123) reported abdominal pain. Of whom 44% used a treatment for pain management	*n*/A
Graff (2010) [36]	*n* = 16 pwCF (age 7–11 y)	AP, flatulence and stool frequency and consistency	Effects of pancrealipase and AS	Symptoms of EPI were less severe and remained relatively stable during pancrealipase treatment	yes
Munck (2012) [37]	*n* = 8 pwCF (age 9–17 y)	28-day symptom diary on bouts of pain incl. 5 questionnaires ^1^	Recurrent AP	Most frequent locations: epigastric region and right or left iliac fossa. *n* = 5/8 pwCF had severe pain intensity at initial visit. *n* = 6/10 AP ≥30 min	*n*/A

PROM: Patient-Reported Outcome Measure, PERT: Pancreatic Enzyme Replacement Therapy, pwCF: people with cystic fibrosis, HRQL: Health-Related Quality of Life, FPS: Face Pain Scale, VAS: Visual Analogue Scale, LS: Lykert Scale, y: years, *n*/A: not applicable, EPI: Exocrine Pancreatic Insufficiency, GI: gastrointestinal, MSAS: Memorial Symptom Assessment Scale. ^1^ Assessing bouts of pain plus five questionnaires: Eland Pain location, pain intensity measured by Faces Pain Scale-Revised, Mc Gill emotional status, Revised Children’s Manifest Anxiety Scale anxiety score, and health-related quality of life (CF-QOL).

## Data Availability

Not applicable.
